# Different culture conditions affect the growth of human tendon stem/progenitor cells (TSPCs) within a mixed tendon cells (TCs) population

**DOI:** 10.1186/s40634-017-0082-8

**Published:** 2017-02-28

**Authors:** M. Viganò, C. Perucca Orfei, A. Colombini, D. Stanco, P. Randelli, V. Sansone, L. de Girolamo

**Affiliations:** 1 Orthopaedic Biotechnology Lab, IRCCS Galeazzi Orthopaedic Institute, Via R. Galeazzi 4, 20161 Milan, Italy; 2 Department of Biotechnology and Biosciences, University of Milano-Bicocca, Milan, Italy; 3 Department of Drug Sciences, University of Pavia, Pavia, Italy; 4 IRCCS Policlinico San Donato, San Donato Milanese, Milan, Italy; 5 Department of Biomedical Sciences for Health, University of Milan, San Donato Milanese, Milan, Italy; 6 IRCCS Galeazzi Orthopaedic Institute, Milan, Italy; 7 Department of Biomedical Sciences for Health, University of Milano, Milan, Italy

**Keywords:** FGF, Tendon, Tendon cells, Clonal selection, Progenitor cells, Tenocytes

## Abstract

**Background:**

Tendon resident cells (TCs) are a mixed population made of terminally differentiated tenocytes and tendon stem/progenitor cells (TSPCs). Since the enrichment of progenitors proportion could enhance the effectiveness of treatments based on these cell populations, the interest on the effect of culture conditions on the TSPCs is growing.

In this study the clonal selection and the culture in presence or absence of basic fibroblast growth factor (bFGF) were used to assess their influences on the stemness properties and phenotype specific features of tendon cells.

**Methods:**

Cells cultured with the different methods were analyzed in terms of clonogenic and differentiation abilities, stem and tendon specific genes expression and immunophenotype at passage 2 and passage 4.

**Results:**

The clonal selection allowed to isolate cells with a higher multi-differentiation potential, but at the same time a lower proliferation rate in comparison to the whole population. Moreover, the clones express a higher amounts of stemness marker *OCT4* and tendon specific transcription factor Scleraxis (*SCX*) mRNA, but a lower level of decorin (*DCN*). On the other hand, the number of cells obtained by clonal selection was extremely low and most of the clones were unable to reach a high number of passages in cultures.

The presence of bFGF influences TCs morphology, enhance their proliferation rate and reduce their clonogenic ability. Interestingly, the expression of CD54, a known mesenchymal stem cell marker, is reduced in presence of bFGF at early passages. Nevertheless, bFGF does not affect the chondrogenic and osteogenic potential of TCs and the expression of tendon specific markers, while it was able to downregulate the *OCT4* expression.

**Conclusion:**

This study showed that clonal selection enhance progenitors content in TCs populations, but the extremely low number of cells produced with this method could represent an insurmountable obstacle to its application in clinical approaches. We observed that the addition of bFGF to the culture medium promotes the maintenance of a higher number of differentiated cells, reducing the proportion of progenitors within the whole population. Overall our findings demonstrated the importance of the use of specific culture protocols to obtain tendon cells for possible clinical applications.

## Background

Tendon cells (TCs) represent 5% of tendon tissue weight and are the main responsible for the maintenance of tissue homeostasis. The majority of these cells (90–95%) consists in a mixed population made of terminally differentiated tenocytes, representing the predominant cell type, and tendon stem/progenitor cells (TSPCs), which show several features of stem cells (Bi et al. 2007; Lui et al. 2011); the remaining 5–10% is represented by chondrocytes, synoviocytes and vascular endothelial cells (Kannus 2000). The possibility to use TCs, and specifically TSPCs, in regenerative medicine approaches is currently under investigation, with promising preliminary results expecially deriving from pre-clinical models. Their application ranges from injective treatment (Ni et al. 2012; Chen et al. 2012) to the use of cell-seeded scaffolds as a form of surgical augmentation (Cao et al. 2002; Chen et al. 2007; Stoll et al. 2011; Chen et al. 2011). To date, due to the absence of specific cell markers suitable for the sorting of tendinous subpopulations containing cells at different stages of differentiation, culturing tendon cells at low density represents the most used method to isolate specific progenitor subpopulations. Although their real effectiveness is still to be clearly demonstrated, several studies have reported satisfactory results using both low density culture and single colony harvesting to isolate TSPCs from a mixed tendon cell population in rat and rabbit (Lui et al. 2011, Zhang et al. 2010, Bi et al. 2007, Rui et al. 2010). Nevertheless, how the culture conditions can influence the TCs population features and its enrichment in progenitor content is still debated, at least for what concern human-derived cells. Different growth factors have been also used in order to enhance or reduce the proportion of either terminally differentiated tenocytes or tendon stem/progenitor cells within TCs culture. TGF-β, IGF-1, BMPs and PDGF were found to be able to induce tenocyte proliferation and, at the same time, to enhance the expression of tendon markers (Gaspar et al. 2015). In addition, IGF-1 could enhance the stem properties of TSPCs in culture (Hollyday C et al. 2016), whereas basic fibroblast growth factor (bFGF) is known to enhance proliferation of fibroblasts (Yun et al. 2010) and to maintain tendon markers expression in cultured tenocytes as well as to enhance their proliferation in combination with PDGF or IGF-1 (Caliari et al. 2013; Qiu Y et al. 2006; Costa et al. 2006).

In this study we have compared different culture conditions of human TCs, assessing their possible influences on the stemness properties and phenotype specific features of tendon cells. In particular, we focused on the use of bFGF and clonal isolation, with the aim to investigate if these approaches will allow to increase the number of tendon progenitors having a translational potential for regenerative medicine applications.

## Methods

### Tendon cells (TCs) isolation, culture conditions and clonal selection

All the procedures were carried out at Galeazzi Orthopedic Institute (Italy) with Institutional Review Board approval (M-SPER-014.ver7 for the use of surgical waste). The donors gave their written informed consent. Gracilis and semitendinosus tendons were harvested from leftover tissue that would otherwise be discarded of eight donors (six males, two females; mean age 31.1 ± 10.9 years) who underwent anterior cruciate ligament reconstruction. Tendon cells were isolated from fragments by enzymatic digestion (16 h, 37 °C,) with 0.3% w/v type I collagenase (Worthington Biochemical Corp., NJ, USA) in high glucose DMEM (HG-DMEM, Life Technologies, Carlsbad, CA, USA) (Rui et al. 2010; de Girolamo et al. 2013). After the digestion, the samples were filtered through a 100 μm cell strainer (Becton, Dickinson and Co., NJ, USA) and centrifuged (300x g, 5 min). The cells were counted and plated at a density of 5 × 10^3^ cells/cm^2^ in complete medium (CM) consisting of HG-DMEM supplemented with 10% FBS (Sigma-Aldrich, St. Louis, MO, USA), 50 U/mL penicillin, 50 mg/mL streptomycin, 2 mM L-glutamine (all from Life Technologies), and maintained in incubator at 37 °C in a humidified atmosphere with 5% CO_2_. Once they reached 80–90% of confluence, the cells were detached with 0.05% trypsin/0.2% EDTA (Sigma-Aldrich) and plated at a density of 3 × 10^3^ cells/cm^2^ and cultured in CM in absence (TCs-) or in presence (TCs+) of 5 ng/mL bFGF (Peprotech, NJ, USA). The cells were used for the experiments until passage 4 (P4). A clonal selection was also performed on all the 8 cell populations included in this study. Cells at P1 were plated at low density, 50 cells/cm^2^ (Rui et al. 2010), and cultured in CM without bFGF. After 3 weeks of culture, the colonies were detached by 0.05% trypsin/0.2% EDTA using Pyrex® cloning cylinder (Corning, NY, USA). Clones were further expanded at normal density to P4 and the multi-differentiation potential and the gene expression of each colony were investigated. Cells cultured at normal density (5 × 10^3^ cells/cm^2^) were used as controls.

### Morphological evaluation

TCs + and TCs- were daily observed at the optical microscope and their morphology at P2 and P4 was evaluated: cells were fixed in 4% paraformaldehyde solution, nuclei were stained with DAPI (1 μg/ml, Life Technologies) and F-actin filaments were stained with Phalloidin (6.6 μM, Life Technologies). The samples were then imaged through a fluorescence microscope (Olympus IX71).

### Doubling time evaluation

The doubling time (DT) of both TCs + and TCs- was recorded from P2 to P4 and calculated according to the following formula: DT = CT/ln(Nf-Ni)/ln2, where CT is the cell culture time (hours), Nf is the final number of cells and Ni is the initial number of cells (Staszkiewicz J et al. 2010).

### Clonogenic ability assay

A colony-forming unit-fibroblast (CFU-F) assay was performed at P2 and P4. TCs + and TCs - were plated in 6 well plate at different seeding density (1 cell/cm^2^; 3 cells/cm^2^; 6 cells/cm^2^; 12 cells/cm^2^; 24 cells/cm^2^; 48 cells/cm^2^) and cultured in CM with 20% of FBS (Lopa et al. 2014). After 14 days, cells were fixed with 4% paraformaldehyde, stained with 2.3% Crystal Violet staining (Sigma-Aldrich) for 10 min at room temperature and then counted. CFU-F frequency was established by scoring the individual colonies composed of at least 30 cells and expressed as a percentage relative to the number of seeded cells.

### Flow cytometry

The immunophenotype of TCs + and TCs- at P2 and P4 was evaluated by Fluorescence-Activated Cell Scanning (FACS) analysis. Cells were washed twice in cold FACS buffer (phosphate-buffered saline without Ca/Mg^2+^, 2% fetal bovine serum and 0.1% NaN_3_). For each sample, 2.5 × 10^5^ cells were single-stained with the following anti-human primary monoclonal antibodies: fluorescein isothiocyanate-conjugated CD90, CD13 and CD45 (Ancell Corp., MN, USA)); biotinylated-conjugated CD34, CD54 and CD105 (Ancell Corp.); and phycoerythrin-conjugated CD73 (Miltenyi Biotec, Germany). Streptavidin–phycoerythrin (Ancell Corp.) was used to reveal the expression. Background fluorescence was set up by negative controls and data (10,000 cell fluorescence events) were acquired using a FACSCalibur™ flow cytometer (BD Bioscences, NJ, USA) and analyzed by CellQuest™ software (BD Bioscences).

### RNA extraction and real time PCR

Gene expression of TCs+, TCs- and clones at P2 and P4 was evaluated by real time PCR (Applied Biosystems® StepOne Plus, Life Technologies). Total RNA was extracted by PureLink® RNA Mini Kit (Life Technologies) and reverse transcripted to cDNA (5 min at 25 °C, 30 min at 42 °C and 5 min at 85 °C) using an iScript™ cDNA Synthesis Kit (Bio-Rad Laboratories, CA, USA). Twenty ng of cDNA were used as template and were incubated with a PCR mix (50 °C for 2 min, 95 °C for 10 min, 40 cycles at 95 °C for 15 s and 60 °C for 1 min) containing TaqMan® Universal PCR Master Mix and Assays-on-Demand Gene expression probes (Life Technologies) for the following genes: *KLF4* (Hs00358836_m1), *OCT4* (Hs04260367_gh), *MKX* (Hs00543190_m1), *DCN* (Hs00370385_m1) and *SCX* (Hs03054634_g1). The fold change in expression was normalized against the expression of the housekeeping gene *GAPDH* (Hs99999905_m1). Two replicates were analyzed for each experimental group. Data were expressed according to the ddCt method.

### Multi-differentiative potential

#### Adipogenic potential

TCs+, TCs- and clones at P4 were seeded in 24-well plates at 10^4^ cells/cm^2^ and differentiated for 14 days with a pulsed adipogenic medium (de Girolamo et al. 2009) consisting of 3 days of induction in CM supplemented with 1 μM dexamethasone, 10 μg/mL insulin, 500 μM 3-isobutyl-1-methylxanthine and 200 μM indomethacin (all from Sigma-Aldrich), followed by 3 days of maintenance in CM supplemented with 10 μg/mL insulin. The cells were fixed in 10% neutral buffered formalin for 1 h and stained with Oil Red O (Sigma-Aldrich) for 15 min to evaluate lipid vacuoles formation. Oil Red O was unstained with 100% isopropanol and then quantified by absorbance at 490 nm (Perkin Elmer Victor X3 microplate reader; Perkin Elmer, Waltham, MA, USA).

#### Osteogenic potential

TCs+, TCs- and clones at P4 were seeded at 10^4^ cells/cm^2^ and differentiated for 14 days in osteogenic medium consisting of CM supplemented with 10 nM dexamethasone, 10 mM glycerol-2-phosphate, 150 μM L-ascorbic acid-2-phosphate and 10 nM cholecalciferol (all from Sigma- Aldrich) (de Girolamo et al. 2009). The extracellular calcified matrix deposition was measured using Alizarin Red-S staining. Cells were fixed with ice-cold 70% ethanol for 1 h and stained with 40 mM Alizarin Red S (pH 4.1; Fluka-Sigma Aldrich, MO, USA) for 15 min. The dye was extracted with 10% cetylpyridinium chloride monohydrate (Sigma-Aldrich) in 0.1 M phosphate buffer (pH 7.0) and the absorbance was read at 550 nm (Perkin Elmer Victor X3 microplate reader).

#### Chondrogenic potential

TCs+, TCs- and clones at P4 were seeded at 10^4^ cells/cm^2^ and cultured for 21 days in chondrogenic medium consisting of HG-DMEM supplemented with 1% of FBS, 2 mM L-glutamine, 50 U/mL penicillin, 50 mg/mL streptomycin, 1 mM sodium pyruvate (all from Sigma-Aldrich), 1% ITS + 1 (1.0 mg/mL insulin from bovine pancreas, 0.55 mg/mL human transferrin, 0.5 μg/mL sodium selenite, 50 mg/mL bovine serum albumin and 470 μg/mL linoleic acid, Sigma-Aldrich), 0.1 mM dexamethasone, 0.1 mM L-ascorbic acid-2-phosphate, 10 ng/ml TGF-β1 (Peprotech) (modified from Barbero et al. 2004). The cells were fixed with 10% neutral buffered formalin solution, rinsed with distilled water, and stained with Alcian Blue solution (pH 2.5) for 30 min (Sigma-Aldrich) to evaluate glycosaminoglycan deposition. The dye was extracted with guanidine hydrochloride (6 M) and the absorbance read at 650 nm (Perkin Elmer Victor X3 microplate reader) (Ruzzini et al. 2014).

### Statistical analysis

Data are expressed as means ± standard deviations. GraphPad Prism v5.0 software (GraphPad Software Inc., La Jolla, CA, USA) was used to perform all the analyses. To assess for adjustment of series of values to normal distribution, the Kolmogorov-Smirnov test was applied. Student’s *t* test was applied to compare values between groups when data were normally distributed, otherwise Mann-Whitney’s test was performed. p values <0.05 were considered as statistically significant. (*p < 0.05).

## Results

### Clones obtained by TCs seeded at low density show a higher multi-differentiation potential and stemness marker expression, but a lower proliferation rate, in comparison to the whole population

Among the 8 TC populations seeded at low density (50 cell/cm^2^), only three gave rise to clones and allowed to proceed with the further experiments. Due to the low number of samples, no statistical analysis was performed, and all following observations refer to trends.

From each of these, a range of 3–11 clones were isolated, with 6.3 ± 4.9 × 10^3^ cells per clone at P2. At P3 the number of cells increased to 4.8 ± 4.2 × 10^4^, even if only the 72 ± 27% of clones survived. At P4, this proportion decreased to 21 ± 29%, with a mean cell count of 1.1 ± 0.6 × 10^4^ cells per clone.

The osteo-differentiated clones showed an increase of +36% in matrix deposition with respect to whole population. Similarly, an increase in their chondrogenic ability was observed in chondro-differentiated clones with respect to whole population (+102%). Indeed, each clone demonstrated extremely different features in terms of differentiation potential, ranging from great to none ability to produce calcified matrix or glycosaminoglycans.

Just one population over the 8 tested allowed a suitable cell harvesting for mRNA extraction. We obtained six different clones from this population, and they showed a quite variable relative expression in all analyzed markers, with no particular correlation with their differentiation ability. Interestingly, the mean relative expression of *OCT4* and *SCX* in the clones resulted 6-fold higher than the whole population, while a 10-fold decrease was observed for what concern *DCN*. Moreover, a positive correlation was found between the expression of *OCT4* and *SCX* among clones of the same population (Pearson’s R^2^ = 0.89, *p* < 0.05), while a negative correlation was observed comparing *SCX* and *KLF4* (Pearson’s R^2^ = −0.91, *p* < 0.05).

### The presence of bFGF influences proliferation rate, clonogenic ability, the expression of CD54 and OCT4

**Fig. 1:**
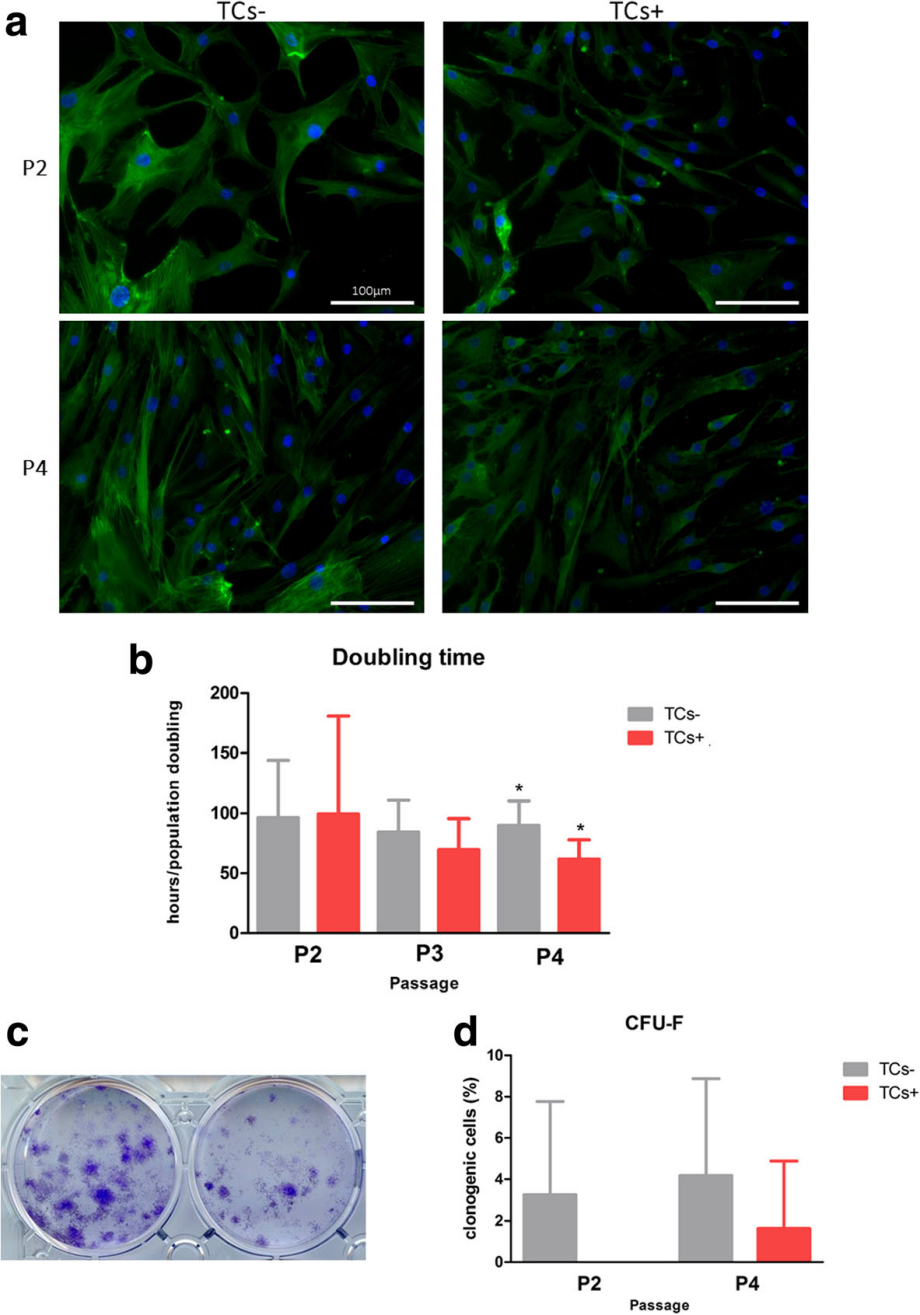
Morphology and clonogenic potential. **a** Cell morphology at P2 and P4, after DAPI/phalloidin staining (scale bars 100 μm). **b** Proliferation ability during passages in culture expressed as mean of doubling times. Levels of significance: *p < 0.05 **c** Representative micrographs of CFU-F at P4. **d** Percentage of clonogenic cells at P2 and P4 (*n* = 4)

TCs cultured in presence or absence of bFGF reveal similar morphology, even if a more pronounced fibroblast-like morphology was observed when cultured in presence of bFGF (Fig. 1a). As expected, the proliferation rate was higher in presence of bFGF, with a doubling time of 70 ± 26 and 62 ± 16 h at P3 and P4, respectively (−18 and −31% vs TCs-, *p* < 0.05) (Fig. 1b). On the other hand, TCs- showed a higher clonogenic ability both at P2 and P4 3.2% ± 2.3% and 4.2% ± 1.6%, respectively) in comparison with TCs + (0% ± 0.0%and 1.6% ± 1.1%) (Fig. 1c, d).

Cell surface markers analysis was performed during expansion (P2 and P4). The presence of bFGF did not influence the percentage of cells positive for the stemness-related markers CD13, CD73, CD90, as well as the percentage of cells negative for CD34, CD45 expression, which are known to be hematopoietic cell markers. The only difference between TC+ and TC- was observed in CD54 expression, which was significantly higher expressed in TCs- at both P2 and P4 in comparison with TCs + (*p* < 0.5). For both the cell types, an increasing trend of CD54 expression from P2 to P4 was observed. The complete data concerning cell surface markers expression are shown in Table 1.

**Table 1:** Cell surface markers expression

	Surface markers	CD13	CD34	CD45	CD54	CD73	CD90	CD105^a^
TCs -	P2	99.7% ± 0.1%	3.7% ± 1.9%	2.6% ± 1.6%	85.7% ± 6.5%*	99.8% ± 0.1%	99.1% ± 0.7%	98.3% ± 1.1%
	P4	99.9% ± 0.1%	1.7% ± 0.7%	0.8% ± 0.7%	92.1% ± 3.0%*	99.9% ± 0.1%	96.8% ± 0.5%	98.4% ± 0.5%^a^
TCs +	P2	99.7% ± 0.1%	2.9% ± 3.2%	0.8% ± 0.7%	46.6% ± 29.2%	99.7% ± 0.4%	99.0% ± 0.5%	98.2% ± 0.7%
	P4	99.7% ± 0.4%	2.7% ± 1.5%	0.4% ± 0.3%	82.5% ± 9.6%	99.8% ± 0.4%	98.6% ± 0.2%	98.6% ± 0.3%^a^

Percentage of positive TCs- and TCs + for the whole panel of surface markers tested at P2 and P4 (*n* = 4)

* *p* < 0.05 TCs- vs TCs+

^a^Data at passage 5

Both TCs + and TCs- cultured for 14 days in adipogenic medium showed no appreciable intracellular lipid vacuole production in comparison with cells maintained in non-inductive medium (data not shown).

**Fig. 2:**
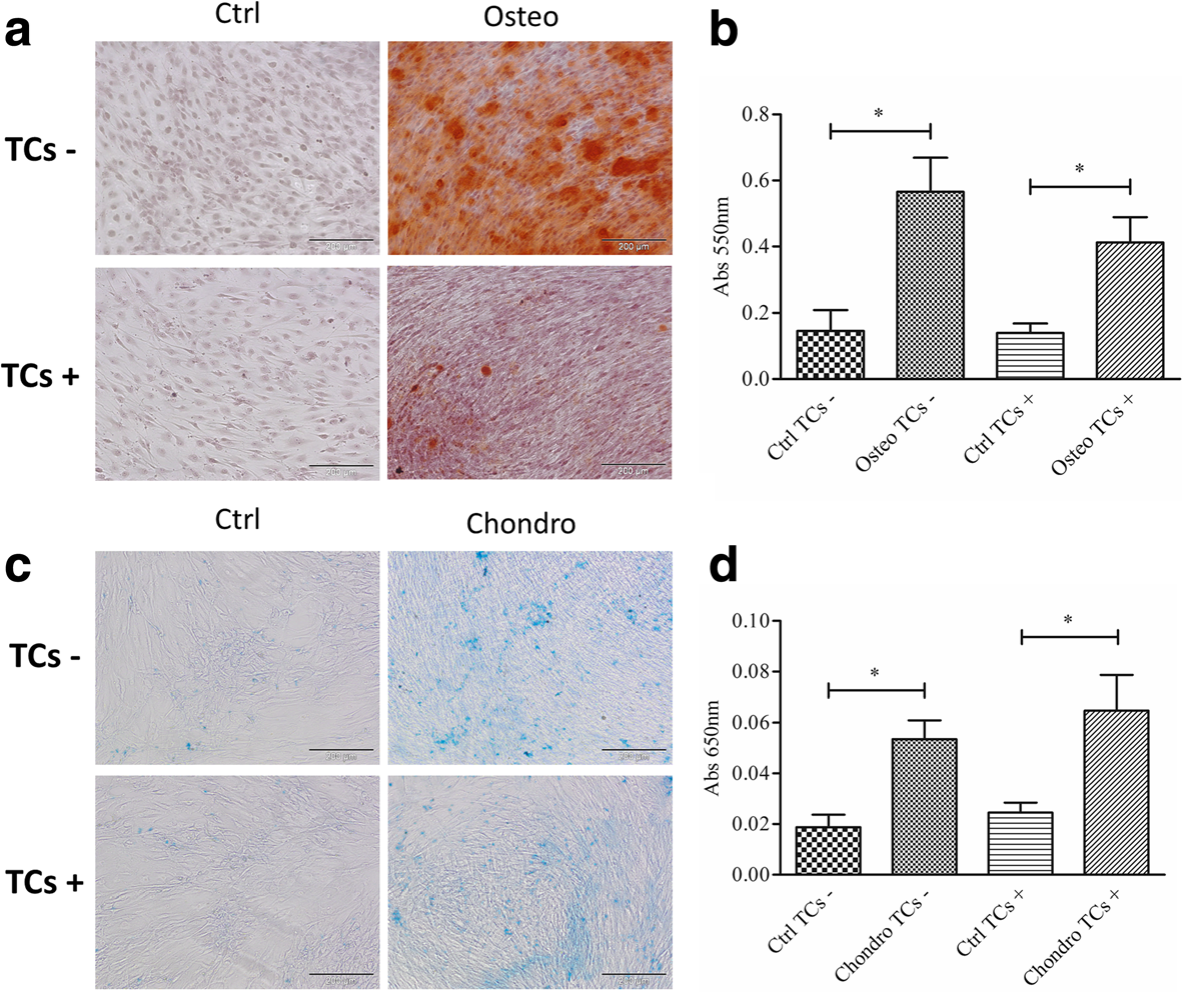
Multi-differentiative potential. **a** Osteogenic differentiated TCs- and TCs + after *Alizarin Red* staining (scale bars 200 μm). **b** Quantification of calcified matrix by AR-S staining and extraction in undifferentiated (Ctrl) and osteogenic-differentiated (Osteo) cells (*n* = 6). **c** Chondrogenic differentiation of TCs- and TCs + after Alcian *Blue* staining (scale bars 500 μm). **d** Quantification of glycosaminoglycans deposition by Alcian *Blue* staining and extraction in undifferentiated (Ctrl) and chondrogenic-differentiated (Chondro) cells (*n* = 4). Levels of significance: * *p* < 0.05

Both in presence or absence of bFGF, TCs were able to differentiate towards the osteogenic lineage. Indeed, after 14 days of culture in osteogenic medium the deposition of calcified matrix was significantly higher in differentiated cells with regards to controls (3.9 fold in TCs- and 3.0 fold in TCs + *p* < 0.05, Fig. 2a, b), with no differences between culture conditions. At the same manner, the chondrogenic potential measured after 21 days of culture in chondro-inductive condition, was similar between the two populations with significantly higher amount of glycosaminoglycans in comparison with the respective controls (2.9 fold in TCs- and 2.6 fold in TCs+, *p* < 0.05, Fig. 2c, d). Interestingly, the basal level of GAG and calcified matrix deposition does not differs between the two populations, also in non-inductive conditions.

**Fig. 3:**
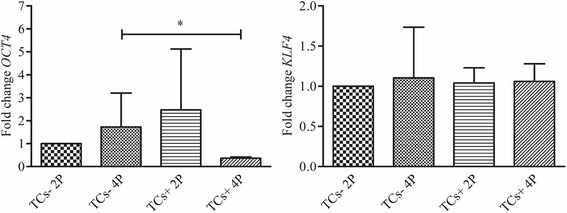
Gene expression of stemness markers. *OCT4* and *KLF4* genes expression in TCs- and TCs + at P2 and P4 normalized to *GAPDH* (*n* = 6). Levels of significance: * *p* < 0.05

A significant decrease of 1.36 fold (*p* < 0.05) in the expression of *OCT4*, was observed in TCs + in comparison with TCs- cultured for 4 passages. Moreover, the cells cultured in presence of bFGF showed a clear decrease in *OCT4* expression from P2 to P4, even if this difference was not statistically significant. On the contrary, *KLF4* expression was stable in both the cell culture conditions and during passages (Fig. 3).

**Fig. 4:**
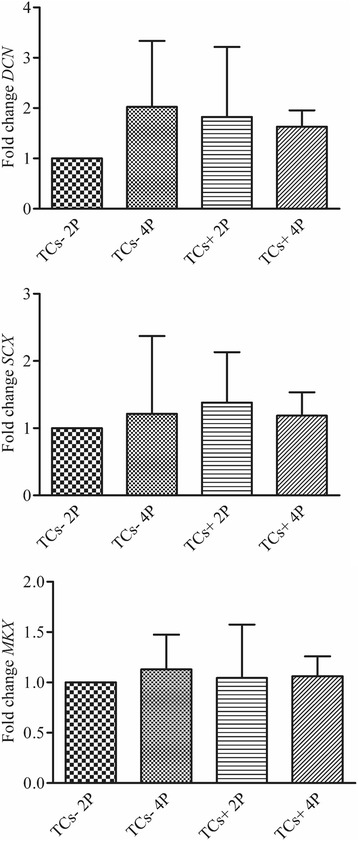
Gene expression of tendon markers. *MKX*, *DCN* and *SCX* genes expression in TCs- and TCs + at P2 and P4 normalized to *GAPDH* (*n* = 6)

The expression of *MKW*, *DCN* and *SCX* was measured at P2 and P4. Slight and no statistically significant differences were observed between TCs- and TCs+. In particular the TCs- expression of *DCN* increased with time in culture, and the presence of bFGF up-regulated its expression as well (Fig. 4).

## Discussion

Adult mesenchymal stem cells represent a tool for clinical applications in regenerative medicine (Ménard et al. 2013). In this field, the use of MSCs for the treatment of tendon disorders is still far from the clinical practice due to the lack of knowledge about the tenogenic potential of the MSCs. Therefore, since the description of tendon stem/progenitor cells in 2007 by Bi and colleagues (Bi et al. 2007), the possible use of autologous tendon cells in tendon regenerative medicine approaches is subject of a growing interest (Ho et al. 2014). Nevertheless, the presence of different subpopulations among the tendon resident cells represents a largely unexplored field of investigation. The lack of a specific terminology and the difficulties in purifying, expanding and maintaining the different cellular subsets are the main obstacles in this field (Docheva et al. 2015). In view of future applications of regenerative approaches for the treatment of tendon disorders, it would be crucial to define the most suitable culture conditions to isolate the different sub-populations within tendon cells, and to improve their ability in promoting tendon healing and regeneration. For this purpose, different culture conditions have been proposed to enrich in vitro cultures with one population or the other, as the application of specific growth factors or patterned substrates, such as tendon derived matrix (Zhang et al. 2011) to simulate the features of the native microenvironment of tenocytes (Gaspar 2015).

In our study we investigated the presence of Tendon Stem/Progenitor Cells (TSPCs) within the tendon resident cells (TCs) isolated from human gracilis and semitendinosus tendons, and we assessed the effects of bFGF and clonal selection as possible strategies to modulate their predominance in culture.

Almost 25% of the TC populations analyzed gave rise to few clones when cultured in clonal selection conditions. Indeed, the low content of progenitor cells in tendons is a well known limitation to their application (Bi et al. 2007). The clones showed a higher mean ability to differentiate toward osteogenic and chondrogenic lineages, as revealed by the production of calcified matrix and glycosaminoglycans, respectively, in comparison with the corresponding whole population, with high differences in the performance of each clone though. Interestingly, the stemness marker *OCT4*, as well as the early tenogenic marker *SCX*, were up-regulated in clones with regards to the whole population, and the expression of these two genes was positively correlated. On the contrary, the late tenogenic marker *DCN* was less expressed in clones in comparison with whole population. Moreover, the expression of the stemness marker *KLF4* resulted slightly higher in clones with respect to the cells cultured at normal density, in particular in presence of bFGF. Nevertheless, the *KLF4* pattern of expression could vary between different conditions, such as stages of cell differentiation, and this could explain the negative correlation between this marker and *SCX* (Zhang et al. 2010).

As expected, all these data showed that the selected clones exhibited a more undifferentiated phenotype in comparison with whole TC population. Despite these encouraging data, the low number of cells that can be isolated by clonal selection make this approach hardly applicable to cell-based therapy, until the identification of growth factors able to enhance the cell yield from isolated clones. The use of bFGF would help reaching this goal, but since it resulted inefficient for the stemness maintenance, we would not recommend it for this purpose. Indeed, bFGF is often used to maintain the cell multipotency in many cells types, and, specifically in tendon cell populations, to enhance the tendon lineage differentiation (Tsutsumi et al. 2001; Hankemeier et al. 2005; Tokunaga et al. 2015). However, its role in promoting the predominance of tendon progenitor cells within the whole tendon cell population have not been investigated so far.

This growth factor, known to enhance cell proliferation in many cell types (Ornitz et al. 2015), exerted the same effect on TCs. The evaluation of the immunophenotype of TCs treated or not with bFGF, showed no difference in term of CD13, CD73, CD90, CD34 and CD45 expression. On the contrary, starting from early passages until passage 4, the expression of the adhesion molecule CD54 (Intercellular Adhesion Molecule-1, ICAM-1) was higher in TCs cultured in absence of bFGF in comparison with TCs+. CD54 has been characterized as one of the peculiar mesenchymal stem cell markers (Calloni et al. 2013) and it is important in inhibiting the osteogenic differentiation of mesenchymal stem cells (Xu et al. 2014). The higher expression of CD54 in TCs- in comparison with TCs + and the increase from passage 2 to passage 4 of its expression in both the populations could indicate a progressive enrichment of progenitors in tendon cells during culture, particularly when maintained in absence of bFGF.

Accordingly, for what concern stemness features, TCs- showed a higher clonogenic ability and *OCT4* expression in comparison with TCs+. Since this gene is known to have a role in sustaining self-renewal capacity of adult stem cells (Niwa et al. 2007), the higher expression in TCs- even at the latest passage (P4) in comparison with TCs+, suggest that the lack of bFGF allowed for the maintenance of a more undifferentiated cell phenotype. The assessment of the multilineage differentiation of TCs in term of adipo, chondro- and osteogenic potential confirmed the findings of a previous study (Stanco et al. 2015).

Indeed, our study showed that both TC- and TCs + had no appreciable adipogenic potential, but they were able to deposit a consistent amount of calcified matrix and glycosaminoglycans in comparison with control cells, with no appreciable differences between them. Similarly, the expression of tendon markers such as *MKW*, *DCN* and *SCX* was not influenced by the presence of bFGF. A limitation of the present work is represented by the low number of cells obtained by clonal selection, resulting in restrictions to the possible analysis. Moreover, the lack of a specific marker of tendon progenitor would not allow the application of cell sorting for the enrichment of the progenitor population. Further studies to investigate this aspect will be crucial for the development of the cell-based therapies for tendon regeneration.

## Conclusion

In conclusion, our study highlights the importance of modulating different culture protocols to obtain useful tendon cells for clinical application. In particular, the addition of bFGF to the culture media caused a loss of stemness features such as clonogenic ability and OCT4 expression, without causing the loss of the tenogenic phenotype. On the other hand, in order to obtain a greater amount of tendon progenitor cells the use of bFGF is not suggested.

Taken together our results showed how much the different cell populations within the tendon tissue are sensitive to the biochemical environment. Further in vitro as well as pre-clinical studies are needed to better correlate the use of different stimuli to the cell responsiveness. Moreover, since tendons are known to be greatly affected to mechanical stress and forces, it would be interesting to identify a possible synergistic effect of biochemical with physical factors.
